# ‘Groping through the fog’: a metasynthesis of women's experiences on VBAC (Vaginal birth after Caesarean section)

**DOI:** 10.1186/1471-2393-12-85

**Published:** 2012-08-21

**Authors:** Ingela Lundgren, Cecily Begley, Mechthild M Gross, Terese Bondas

**Affiliations:** 1 Institute of Health and Care Sciences at the Sahlgrenska Academy, University of Gothenburg, Box 457, S-405 30, Gothenburg, Sweden; 2 School of Nursing and Midwifery, Trinity College Dublin, Dublin 2, Ireland; 3 Midwifery Research and Education Unit, Hannover Medical School, Hannover, Germany; 4 Faculty of Professional Studies, University of Nordland, Bodo, Norway

**Keywords:** Metasynthesis, Qualitative studies, VBAC, Women's experiences

## Abstract

**Background:**

Vaginal birth after Caesarean section (VBAC) is a relevant question for a large number of women due to the internationally rising Caesarean section (CS) rate. There is a great deal of research based on quantitative studies but few qualitative studies about women's experiences.

**Method:**

A metasynthesis based on the interpretative meta ethnography method was conducted. The inclusion criterion was peer-review qualitative articles from different disciplines about women's experiences of VBAC. Eleven articles were checked for quality, and eight articles were included in the synthesis.

**Results:**

The included studies were from Australia (four), UK (three), and US (one), and studied women's experience in relation to different aspects of VBAC; decision-making whether to give birth vaginally, the influence of health professionals on decision-making, reason for trying a vaginal birth, experiences when choosing VBAC, experiences of giving birth vaginally, and giving birth with CS when preferring VBAC. The main results are presented with the metaphor *groping through the fog*; for the women the issue of VBAC is like being in a *fog,* where decision-making and information from the health care system and professionals, both during pregnancy and the birth, is unclear and contrasting. The results are further presented with four themes: ‘to be involved in decision about mode of delivery is difficult but important,’ ‘vaginal birth has several positive aspects mainly described by women,’ ‘vaginal birth after CS is a risky project,’ and ‘own strong responsibility for giving birth vaginally'.

**Conclusion:**

In order to promote VBAC, more studies are needed from different maternity settings and countries about women's experiences. Women need evidence-based information not only about the risks involved but also positive aspects of VBAC.

## Background

The number of women with Caesarean section (CS) in their history is related to a high and rising CS-rate in an international perspective; for example, CS-rates rose in Sweden from 5% in the beginning of the 1970s to 17.2% in 2007 [1], in UK from 9% in 1980 to 25% in 2003 [2], and in Ireland from 11.8% in 1991 to 27% in 2009 [3]. Today the CS-rate is 15.1% in Netherlands [4], 17.1% in Finland [4], 28% in Australia [5], 32.7% in Taiwan [6], and 32.7% in Germany [7].

Due to the rising CS-rate a large group of women and health professionals have to consider the choice between an elective CS or vaginal birth (VBAC) in subsequent birth, a decision which should be individually based [8,9]. VBAC is recommended as safe and as best practice for the majority of women [10,11], is associated with lower maternal mortality than repeat CS, and less overall morbidity for mothers and babies [11]. Similar to the CS-rate, VBAC-rates differ internationally. In Ireland, Germany and Italy the VBAC-rate is 29–36% compared to 45–55% in Netherlands, Sweden and Finland [4]; in the the United States it is 10.1%, and in Australia 19%, and has declined over time [9]. VBAC guidelines from UK, Australia, New Zealand, Canada, and the US are characterized by quasi-experimental evidence, which led to wide variability in clinical practice [12].

The perspective of pregnant women regarding birth risks in a subsequent pregnancy following prior CS are not well understood [13]. There is a great deal of research based on quantitative approaches on VBAC but very few qualitative studies about women's experiences [5,14]. Studies have focused on women's perspective of decision-making in relation to mode of delivery in the subsequent birth after a previous CS. Women experience decisional conflicts and uncertainty and need individual and structured information [6,10,15-17]. Studies focusing on women's experiences of giving birth vaginally after a previous CS birth shows that they express a belief in the importance of a natural birth [18,19], and that psycho-social dimensions that go beyond the birth are of importance for them [20].

In summary, VBAC is a phenomenon relevant for a large group of women due to the rising CS-rate. There is a need to integrate qualitative findings of women’s experiences of VBAC to influence evidence-based practice but also to generate new research questions [21-24]. The objective of this metasynthesis is therefore to integrate the findings and deepen the understanding of women’s experiences of VBAC.

## Method

This metasynthesis was based on the interpretative meta ethnography described by Noblit and Hare [21]. The synthesis is focused on creating new knowledge and it is based in interpretation, and not aggregation [22,24,25].

The challenge is to find, classify and integrate findings from qualitative studies using multiple methods from several epistemological and theoretical perspectives [26]. Each study was characterized according to authors, discipline, method, theoretical perspective, data collection, setting, and aim (Table 1). The subject is the interpretation of findings and does not use primary datasets. The core is translation, by which is meant the interpretation of findings from different studies that share similar research questions [21].

**Table 1:** Articles included in the metasynthesis and quality assessment

**Reference**	**Aim**	**Method**	**Data collection Setting**	**Quality assessment**
**Author**		**Theoretical**		
**Discipline**		**perspective**		
2. Emmet, Shaw, Montgomery, Murphy, Nursing	To explore women's experiences of decision-making about mode of delivery after previous CS	Qualitative study	21 women with a previous CS	M:36
		Framework approach	12 planned a VBAC, 9 planned a CS	
			The participants home	
			Two city hospitals England and Scotland	
13. McGrath, Phillips, Vaughan	To explore the decision-process from the mothers' perspective with regard to subsequent birth choice for women who had previously been delivered by CS	Descriptive phenomenology	4 women who had a VBAC	M:34
Nursing		Van Manen	Locations of the participants' choice 6-8 weeks post partum	
			Australia	
18. Phillips, McGrath, Vaughan	The reasons motivating women to try for a VBAC from the perspective of women	Descriptive phenomenology	4 women who had a VBAC	M:35
Nursing		Van Manen	Locations of the participants’ choice 6-8 weeks post partum	
			Australia	
19. Fenwick, Gamble Hauck Midwifery	Explore and describe the childbirth expectations knowledge, beliefs and attitudes of women who have experienced a CS and would prefer a VBAC in subsequent pregnancy	Thematic analysis	35 women recruited from 157 respondents; 24 who attempted a vaginal birth and 11 who would choose this in a subsequent pregnancy	M:36
			Australia	
20. Meddings, Phipps	The lived experience of women who elected to attempt a vaginal birth following a previous CS	Phenomenological method	8 women recruited via community	M:31
Haith-Cooper, Haigh Nursing			Pregnancy 34 weeks and 6 weeks after birth	
			Participants' own home UK	

29. McGrath, Phillips	The focus is on women who valued a vaginal birth who delivered by CS	Descriptive phenomenology	8 women who valued a vaginal delivery but who delivered by CS	M:34
Vaughan			Locations of the participants' choice	
Nursing			6-8 weeks post partum	
			Australia	
30. Goodhall, McVittie,	Explore mother's perceptions of the influence of health professionals (GP, midwives, and consultants) on decisions as to mode of delivery of second children, following a previous CS.	Interpretative phenomenology	10 pregnant women (medium gestation of 32 weeks) recruited via Edinburgh	M:32
Magil				
Psychology			National Childbirth Trust and personal contacts	
			Interviewee's home	
			UK	
31. Ridley, Davis	Discover what influences women in the decision to deliver via VBAC	Descriptive qualitative method	4 women delivered via VBAC	M:35
Bright, Sinclair			2-4 months post partum	
Nursing			Postpartum unit in a hospital	
			US	

M = Moderate quality.

In the analysis process the preservation of meaning from the original text was important. The articles were independently reviewed and read through several times to get a grasp of the whole and to categorize them using the key themes, categories, metaphors, phrases, ideas, and concepts in the findings of the study. The themes were systematically juxtaposed to identify homogeneity and to note if there was discordance or dissonance between the themes. We explored the convergence of the themes across the articles. In the last phase the themes were synthesized. The findings were seen as analogous and compatible between the studies [21].

### Sampling, inclusion and exclusion criteria

The inclusion criteria for the studies were peer-reviewed empirical qualitative studies in different disciplines in English from women’s perspectives of VBAC. The study includes research published between the years 2002–2010. No studies were found before 2002. The exclusion criteria were studies that were quantitative in design and included mixed studies, and mixed events and time period where it was not possible to separate findings related to VBAC.

**Figure 1:**
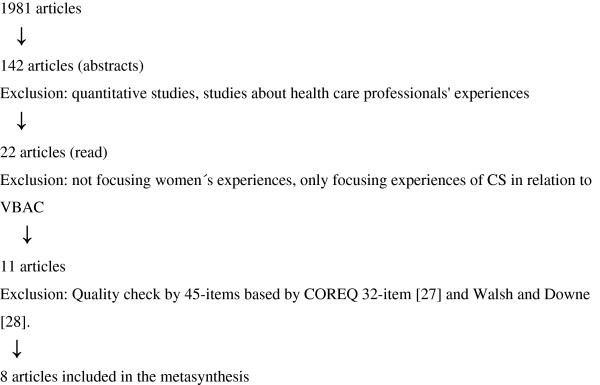
Flow chart summarizing search strategy

Health care related databases were searched in different disciplines and findings from different cultures with the chosen keywords. Previous literature reviews were searched, and author and ancestry search was performed to access studies not identified through the database search. The following databases were searched: CINAHL, EBSCO, Journals@OVID, Pubmed, PSYCHINFO, using the keywords VBAC, vaginal birth after caesarean section, qualitative study, experiences, qualitative and women's experiences in various combinations. In total, 1981 papers were identified; of these, 1959 were excluded after reading the title or abstract, when it became apparent that the paper did not fit the inclusion criteria. The remaining 22 papers were obtained and reviewed in full text format. Eleven were excluded at this stage, as not focusing on women's experiences, or only focusing on experiences of CS in relation to VBAC (Figure 1).

The final 11 papers were assessed for quality, initially using the COREQ 32-item check-list [27]. However, it was noted that the COREQ tool did not include some aspects that have been identified as important in qualitative research, such as ethical issues, thorough use of the literature, quality and audit mechanisms, relevance and transferability. Accordingly, we incorporated 13 other items into the check-list (items 9, 10, 12, 13, 33–35, 40–45) and adapted four items (items 8, 26, 28, 29) relating to these aspects (Table 2), derived from the work of Walsh and Downe [28]. We found this composite grid to be useful in determining the quality of the papers and assisting the decision for inclusion or exclusion.

**Table 2:** Quality assessment

	
**Domain 1: Research team and reflexivity**	
1.	Statement of which author/s conducted the interview or focus group*
2.	List of the researchers’ credentials, e.g., PhD, MD*
3.	Statement of their occupation at the time of the study*
4.	Indication of the gender of the researcher(s)*
5.	Statement of relevant experience or training that researcher(s) had*
6.	Statement of any relationship established between participants and researchers prior to study start*
7.	Statement of participant knowledge of the interviewer*
8.	Evidence of self-awareness/insight in the characteristics reported about the interviewer/facilitator: e.g., assumptions, bias, reasons for or interest in the research topic*
Domain 2: Scope and purpose*	
9.	Link between research and existing knowledge demonstrated*
10.	A clear aim for the study was stated*
**Domain 3: study design**	
11.	A clear methodological orientation was stated to underpin the study e.g. grounded theory, discourse analysis, ethnography, phenomenology, content analysis*
12.	Ethical committee approval granted*
13.	Documentation of how autonomy, consent, confidentiality etc. were managed*
14.	Description of how participants were selected: e.g. purposive, convenience, consecutive, snowball*
15.	Description of method of approach e.g. face-to-face, telephone, mail/email*
16.	Sample size: number of participants in the study declared*
17.	Number of people who refused to participate or dropped out given, with reasons*
18.	Description of setting of data collection e.g. home, clinic, workplace*
19.	Declaration of presence of non-participants, if applicable*
20.	Description of important characteristics of the sample e.g., demographic data, date data collected*
21.	Description of interview guide given e.g., questions, prompts, guides, and any pilot testing*
22.	Number of repeat interviews given, if applicable*
23.	Statements of audio/visual recording or not*
24.	Statements of whether or not fields notes were used*
25.	Duration of interviews or focus group given*
26.	Evidence provided that the data reached saturation or discussion/rationale if they did not*
27.	Statements of whether or not transcripts were returned to participants for comment and/or correction*
**Domain 4: analysis and findings**	
28.	Number of data coders given/evidence of more than one researcher involved*
29.	Description provided of the coding tree/discussion of how coding system evolved*
30.	Statement of whether themes were identified in advance or derived from the data*
31.	Statement of manual analysis, or the software that was used to manage the data*
32.	Statement of whether or not participants provided feedback on the findings*
33.	Statements of whether or not deviant data were sought, if applicable*
34.	Statement of whether or not researchers “dwelt with the data”, interrogating if for alternative explanations of phenomena*
35.	Sufficient discussion of research processes such that others can follow ‘decision trail’*
36.	Identified participant quotations (e.g. by participant number) presented to illustrate the themes/findings*
37.	Consistency seen between the data presented in the findings*
38.	Major themes clearly presented in the findings*
39.	Description given of diverse cases or minor themes*
40.	The results are presented with an essence (phenomenology), main interpretation (hermeneutics), theory/main concepts (grounded theory), main theme (content analysis)*
41.	Evidence of systematic location and inclusion of literature and theory to contextualize findings*
**Domain 5: Relevance and transferability**	
42.	Clearly resonates with other knowledge and experience*
43.	Provides new insights and increases understanding*
44.	Limitations/weaknesses clearly outlined*
45.	Further directions for investigation outlined*

*Yes, no or not applicable

Two authors assessed each study and agreed its inclusion. Eight papers that were deemed to be of medium quality (positive ratings for 31–38 items) were included in this review (Table 1). No papers were marked as high quality (rated positively for 39–45 items) The remaining three papers were excluded due to an overall rating of minor quality (30 or below). The excluded papers had lower ratings in relation to all domains; research team and reflexivity, scope and purpose, study design, analysis and findings, and relevance and transferability.

## Results

The results show that four studies are from Australia [13,18,19,29], three from UK [2,20,30], and one from US [31]. The women's experiences were requested concerning different aspects of the following phenomena: experiences of decision-making - whether to give birth vaginally or with CS during the subsequent birth [2,13,20], experiences of the influence of health professionals on decision-making [30], reason for trying a vaginal birth after a previous CS [18,20,31], experiences when choosing VBAC [18,19,29], experiences during the subsequent birth giving birth vaginally [18,20,31], and experiences with CS during the subsequent birth [13,30]. Experiences of giving birth vaginally [13,18-20,31], and with CS [2,13,18-20,29,31] are described. In one study no information was given about the subsequent birth experience [30]. Interviews with women were performed during pregnancy [19,20,30], and two to eight months after birth [2,13,18-20,29,31]. Altogether 94 women participated in the studies. There are duplicates of participants in three studies [13,18,29].

Experiences of vaginal birth after a previous CS are for women like *groping through the fog,* where decision-making and information from the health care system and professionals, both during pregnancy and the birth, is unclear and contrasting. Being in a *fog* is like groping for a way out by asking health care professionals during pregnancy, and even during the birth, but getting no clear answer, contrasting answers or answers not in agreement with their own choice. Women have to fight for a vaginal birth as, even if the health care system is presenting itself as ‘pro VBAC', the reality they experience is very different. The system can be experienced as supportive in relation to the woman's choice but VBAC is mostly mediated in relation to risks, and information on the positive aspects of giving birth vaginally is seldom given. Thereby the whole project of giving birth vaginally after a previous CS is experienced as *paradoxical*, and thus is like being in a *fog* with no clear view about what is best for the woman as an individual. The clear individual perspective is coming from inside the woman herself as a desire to give birth vaginally. Giving birth vaginally is described as empowering, as best for the baby and as important in a life-perspective for them as women, but not a real choice for some women, which is contrasted to negative previous experiences of CS. Four main themes were seen: ‘Own strong responsibility for giving birth vaginally', ‘Vaginal birth after CS is a risky project', ‘Vaginal birth has several positive aspects mainly described by women', and ‘To be involved in decision about mode of delivery is difficult but important.’ The four themes are presented with sub-themes (Table 3).

**Table 3:** Themes and sub-themes

**Sub-themes**	**Themes**	**Articles**
In relation to the women themselves	Own strong responsibility for giving birth vaginally	13,18,19,29,30
In relation to information		2,13,18,19,29,30
In relation to health-professionals		13,18,19
To have to confront serious risks mediated by health-professionals	Vaginal birth after CS is a risky project	2,13,18,19,29,30,31
Lack of information about the benefits of vaginal birth		2,13,30
Not supported if you want a VBAC		2,13,19,30
Good for the baby and the mother- baby relationship	Vaginal birth has several positive aspects mainly described by women	2,18,19,20,29,30,31
A meaningful experience of importance for them as women		18,19,20,29,30,31
An easier birth in relation to recovery afterwards		2,19,20,31
Some health professionals are pro VBAC		2,13,20,29,31
Not being informed enough	To be involved in decision about mode of delivery is hard and important	2,13,19,20,31
Conflicting information		2,18,19,20,29,30,31
Important to have a choice		2,19,20,29,31
Uncertainty in relation to choice		2,19,20,29,31
Information/support from others not the hospital		2,13,18,19,29,31
Support from professionals		2,13,29,30,31
Experiences from the last birth influence the choice		2,19,29,30,31

### Own strong responsibility for giving birth vaginally

#### In relation to the women themselves

The women described that they had their own personal responsibility in relation to giving birth vaginally after a previous CS [13,18,19,30,31]. This responsibility could be related to the women themselves and their attitudes to birth, expressed as being strongly, deeply and highly motivated [18].

*Oh yes, deeply motivated. … I didn't feel like, if I wasn't deeply motivated it wouldn't have happened*[18], p.81.

#### In relation to information

The women described how they had to seek information to gain knowledge of how to facilitate normal birth [2,13,18,19,30,31], which could sometimes be found by accessing their own medical record from the first delivery [31]. Individual responsibility also means an openness to try a vaginal birth, wanting a natural birth and positive self-talk [18], and experiencing that it is their decision if they want to try a VBAC or not [30].

*(The consultant) said that it was absolutely up to me because there was no particular reason why my last one turned out to be a Caesarean, that it was 50/50 probably that I would need another one again and it was totally up to me if I wanted to try or not*[30], p 8.

#### In relation to health-professionals

Individual responsibility also means a responsibility to communicate with the health professionals [18] with a determined approach [13,18,19]. For some women this can be experienced as being in a bit of a fight [18].

*So I knew that I needed something just to relax me for that interim time. Very prepared, yes. I had to be to do this because I did feel like I was in for a bit of fight near the end*[18], p. 81.

### Vaginal birth after a CS is a risky project

#### To have to confront serious risks mediated by health-professionals

In almost all studies women describe how they predominantly have been informed about the risks involved in giving birth vaginally [2,13,18,19,30,31]. For some women these risks meant that they did not have a choice to give birth vaginally [29]. The risks that the women were informed about were uterine rupture [2,13], death of the child or mother or both [13,19], the risk of ending up having another CS [2,13], and being irresponsible and putting the baby at risk [19,30].

*All those horrible things could be wrong, you could lose your baby, your uterus could rupture, you could bleed to death*[19], p.1565.

The women had to confront serious risks in relation to their decision whether to give birth vaginally or by CS [2,31], in a context of high childbirth intervention rates and a risk focus [19]. Information about risk was mediated by physicians and midwives [2,13,18,19,30,31], but could also be in the form of materials such as a CDROM [31]. The women describe how they were informed about success rates of delivering vaginally, mediated by different percentages (20 -70%) in relation to their individual risks [18,30], and as ‘odds’ being against them [18]. Therefore, for some women, at the back of their mind they were thinking that they probably were not going to be able to give birth vaginally [30]. 

*They examined me and they said it was entirely up to me but they reckoned I didn't have a good chance of having him myself, em, 30%, they said I would probably end up having to have an emergency Caesarean*[30], p.8.

*The information sheet noted that only 20% of women give birth naturally successfully after a Caesarean so I realized that the odds were against me but I was determined anyway*[18], p.81.

#### Lack of information about the benefits of vaginal birth

When health professionals are explaining the risks involved with VBAC, the women were reflecting the fact that no information was given about the benefits of vaginal birth [2,13,30], only the downside and risk [13], which may be experienced as strange [2]. The hospital was experienced as more anxious than the women [2]. For some women, guilt was involved if they wished to have a vaginal birth and were thereby accepting the risks [30]. The women may find some good stories about vaginal birth but miss being given concrete information [30].

*I could insist on trying for a normal delivery but there's the guilt that you're being irresponsible and putting the baby at risk*[30], p.9.

*You know there might be one in a hundred chances that I have a uterine rupture, but they kept focusing on the fact that I might be that. Might be that one person that has it. Me, I was thinking ‘look, I'm most likely going to be one of the 99’*[13], p. 278.

#### Not supported if you want a VBAC

Because vaginal birth may be seen as a risky project by health professionals, women may feel that they are not supported if they state that they want a vaginal birth [2,13,19,30]. Support was lacking both from midwives and doctors [2,13,30]. From the perspective of the hospital, vaginal birth is seen as a risky project [13]. Some women feel considerable pressure from their doctors for CS, which was intensified with comments about being selfish, and putting oneself at risk of uterine rupture and bleeding to death [19]. Powerful medical recommendations could also be made during the birth that influences women, sometimes leading to CS. Some women felt that doctors would let them try but that they would intercede quite quickly [30]. Some women experienced that, although the hospital purported to be pro VBAC, the subliminal messages they were being given all suggested that vaginal birth was unlikely [30].

*I feel every time I go and see the doctor or the midwife they keep talking about elective Caesareans…they keep finding reasons why I'll probably need an elective Caesarean so yeah it feels like choice is a lot more limited this time*[30], p.8.

### Vaginal birth has several positive aspects mainly described by women

#### Good for the baby and the mother-baby relationship

In almost all studies, positive aspects of giving birth vaginally are described by the women [2,18-20,29-31]. Natural birth is described as good for the baby [18-20,31], exemplified with bonding [19,20], the best start for baby [18], for maternal infant-relationship and well-being [19,29], and breast-feeding [20]. Vaginal birth was believed to provide bonding to a much greater extent than CS [19]. Women also said that it is important for the baby to pass through the vagina to aid lung expansion [19]. Vaginal birth was said to improve the emotional contact with the baby [19], and women did not want professionals to take the baby after the birth [31]. Vaginal birth decreases the risk of anything happening to the baby [31], and promotes health and well-being of both mother and baby, enhancing maternal interactions and the transition to motherhood [19].

*By doing the vaginal birth you were really giving your baby a better chance – certainly*[18], p.80.

*I didn't hold her for a week and I didn't want that to happen* (again) [31], p.669.

Normal birth was preferred because it reduce the number of drugs [18,19], and interventions such as epidurals and induction of labour [19], had better delivery outcomes [31], and was safer [31]. To deliver a baby was described as natural [18,19,29], and considered to be a normal but significant life event [18,19], and a feeling of failure was described if not being able to birth vaginally [29].

*To deliver a baby is so natural. That's what it was meant to be about. So when you get told: ‘you're not going to do that, you're going to have yours pulled out of your belly’, it does, it makes you feel ‘oh!’*[29], p.29.

#### A meaningful experience of importance for them as women

Giving birth vaginally was also described as good for the woman, expressed as satisfying and empowering [18], important for women to reach their goals [19], a meaningful maternal experience in life [18,29], and as an integral part of being a mother and a woman [19].

*I felt very empowered. Even more passionate about it than before*[18], p.81.

The women describe that they wanted to experience a natural birth and the function of the female body [18,20,31], by working with the body [19], active participation [19], and giving their body an opportunity to experience natural childbirth [19]. The process of birth was described as when one stage triggers something in the human body and mind to flow on to another stage [31]. Women's bodies were described as designed to give birth vaginally [19,30].

*You're built to have a baby naturally and I would just prefer to do it naturally*[19], p. 1565.

The women want to see what it is like to give birth vaginally [2,19,29,31], which was described as the ultimate birth [29,31], what birth is about [29], and nature's way, the proper way and intended way [19]. The women express a strong maternal drive to give birth naturally [19,31]. Physical and emotional factors are important in relation to giving birth vaginally [31]. Belief systems such as religion could also influence the decision [19]. Some women who valued a vaginal birth but delivered by CS expressed that they would have loved having a vaginal delivery [2,19,29], and regret that they did not try, and they are disappointed or even depressed [29].

*I knew that they would talk about a CS…I didn't want to have one…I wanted to have a vaginal, normal delivery if you want to say that…with a lot more of my input…I wanted to do it, plan it, and do it my way this time…I had a lot more input…a lot of it is control*[31], p.668.

*But you know, like a cesarean ago I would have been: ‘oh, yeah, go for cesarean’. But now that I'm definitely looking down the barrel of not being able to ever have a natural birth now. Actually this is my last. I'm very disappointed*[29], p.30.

#### An easier birth in relation to recovery afterwards

Vaginal birth was also preferred due to easier, shorter and quicker recovery after the birth [2,19,20,31]. It was experienced as easier with a small child at home compared to CS [31]. A vaginal birth was also described as less interrupting to daily life [19]. CS resulted in a more painful and longer recovery [20]. Vaginal birth was also easier in relation to family obligations [20]. Women describe that they can walk after VBAC and do not need to rest so much in the following 6–8 weeks [31]. The inability to drive immediately following CS was also felt as very prohibitive [19,20].

*I'm saying that it would be a lot of hassle after the event, and being in a state with stitches or whatever and being told you can't do this and you can't do that for six weeks…my little boy's at nursery and so it would be difficult if I can't drive to get him to the nursery and all that kind of thing*[20] p.164.

#### Some health professionals are pro VBAC

Even if positive aspects of vaginal birth were mainly described by the women, some studies indicate that health professionals prefer VBAC but this was not explicitly stated, according to the women [2,13,20]. Trying for a normal birth is experienced as preferable [20]. A physician could also be perceived to be against CS [31].

*The doctor was very much against CS*[31], p.668.

### To be involved in decision about mode of delivery is difficult but important

#### Not being informed enough

Women describe lack of information from the health care system [2,13,19,20,30], which negatively influences decision-making about mode of delivery. They describe that they were unprepared for labour [20], ill-informed [2], were lacking knowledge [30], and that information after the first CS would have been helpful [2]. In relation to decision-making they need more facts in order to decide [2,30], and need to know, and be brave enough to ask, the right questions [2]. They also needed more information based on the individual, not on routine [2,30].

*She’s got four children herself and she’s of the opinion . . . ‘Look, if the pain gets too bad, you’ve already had a Caesarean. All the female gynaecologists themselves would just opt straight in and have a Caesarean. That’s what they’re all doing . . . Why go and put yourself through that again if you had such a terrible experience the first time? . . . Look, I’m all for you wanting to try it. We’ll give it a go, if it gets too hard and too bad, straight in for a Caesarean. You don’t need to muck around.’ That was her opinion. I said, ‘Okay. I really want to do it though’*[13], p.279.

#### Conflicting information

Health-care professionals were seen as mediating between conflicting, and sometimes contradictory, information [2,13,20,30]. Women describe how individual doctors have different opinions [2,13] about what is the best choice. They feel that there is a lack of medical consensus as to whether induction should be attempted following a previous CS [2,20]. A personal choice for vaginal birth can be in conflict with clinician’s expectations [30].

*Every time you'd see a different doctor. I don't think I ever saw the same doctor. Some seem to be more towards the vaginal birth than the Caesarean and others the other way around. I think it is a bit of personal preference* really [13], p.277.

#### Important to have a choice

Women describe that it is of importance to have a choice about mode of delivery [2,18-20,30,31]. Health-professionals allowed the women to make decisions [2,18,20,30,31], and it was important to have an opportunity for both vaginal birth and CS [18,29-31]. Involvement in decision-making gave confidence and increased the trust in the carers [20], gave a sense of being active [31], of having choice in relation to interventions [20], and that the choice was completely up to the woman [30]. Being involved in decision-making also gave a sense of control [31], which many women felt was important to retain [19,31]; however, some women in one study felt relief when they relinquished control, as it avoided them feeling guilty over making the decision [18]. Women also expressed respect of others’ decisions [18,19,29].

*I knew I wanted to try it from the beginning and I knew that as soon as I got pregnant… When I went to the doctor's office the first time, I asked to see if I could try it, and they were all really supportive*[31], p.668-669.

#### Uncertainty in relation to choice

Women felt uncertainty in relation to choice [2,19,20,30,31]. Uncertainty may lead to a ‘wait and see-policy’ [2], being ‘back and forward’ [30], and changing their mind several times before the birth [19], and not being strong on standing up for what they really want [30]. Women expressed uncertainty in relation to labour [20,30], particularly the second stage of labour [20]. Some women were anxious about the choice even after the birth [2]. Uncertainty could lead to changing specialist, hospitals and getting a second opinion [19]. For some women the solution was to rely on the professionals ‘who know best’ [20,30].

*I don't know what to think really. It's a bit daunting thinking about having a normal birth after a Caesarean section. I don't know what to expect*[20], p.164.

*I was back and forward. I was quite, on a few occasions I said ‘right, I'll go for the section’ to both the consultant and the midwife. I'm the kind of person that I listen to people's advice. I'm not very strong on right this is what I want*[30], p.9.

#### Information/support from others, not the hospital

Information and support from others, not the hospital, was of importance for the women [2,13,18,19,30,31]. They received information and opinions from their partner, family, friends and relatives [2,19,30,31]. Women read books and got information by internet and television [2,19,30,31], which could be experienced as both helpful and non-helpful. Support also came from women with similar experiences of birth [19].

*I read all the research, read a lot of other women's experiences and I contacted a support group*[19], p.1565.

#### Support from professionals

Support in relation to choice was mediated by doctors, and midwives working at hospitals and in the community [2,13,29-31]. Information was most commonly provided during hospital consultations [2,29-31]. Therefore the decision whether to give birth vaginally or by CS was mostly influenced by doctors [2,29-31], who were experienced as supportive of the women's decision [2,29-31].

*I felt that the choice was mine completely and he just basically said to me there and then ‘we can set a date, the 15*^*th*^*of May, how does that suit you, at 8 o'clock in the morning and I just felt completely flooded with relief*[30], p.9.

#### Experiences from last birth influence the choice

Previous birth experiences influenced women's choice [2,19,29,31]. The CS-experience was for some women connected to disempowerment, being powerless, helpless, angry and ‘ripped off’ [19], and loss of confidence in their body’s ability to give birth [29]. Being separated from the baby was also a negative aspect of the previous birth [19,31]. Further, the birth environment [19] and the relationships with professionals were of importance [19]. Some women express that they will do it differently this time [31], which could mean a vaginal birth or a CS. Some women would not try giving birth vaginally [29], and do not like labour pain [29]. For some, the experience of CS has strengthened their desire to have a normal birth [19], but for others a CS was the choice they must make, connected to a desire to have a healthy baby [19,30], and not as an easy and weaker option [30].

*I think the way people handle that first one either builds confidence or takes away people's confidence*[29], p.29.

## Discussion

This metasynthesis offers qualitative evidence from the women’s perspectives on VBAC to complement and deepen the empirical studies in the field. Women’s experiences were studied from different disciplinary perspectives but qualitative research on VBAC seems to be limited to a few countries, notably in an Anglo-American context. There is always a tension between combining studies and retaining the uniqueness of each study. However, we tried to preserve the significance by remaining close to them, going back and forth in the interpretation in order to not lose sight of the primary study, and use citations [24,26]. The researchers had different cultural and ontological perspectives that enabled a reflective and critical attitude [26].

The main results from our study shows that experiences of VBAC is like *groping through the fog*, where decision-making and information from the health care system and professionals, both during pregnancy and the birth, is unclear and contrasting. These findings are in line with Endozien's [8] statement that there is an unmet need for clinicians to provide sufficient information to women, so that the woman's choice can be an informed one. Further, our metasynthesis shows that women's experiences of VBAC are only studied in an Anglo-American context, as the studies were from US, UK and Australia. This is an interesting finding since the high CS-rate occurs world-wide, and the question about women's experiences should be of interest for other maternity care settings and countries. For example, no studies were found from the Netherlands or Scandinavian countries, which in comparison with other high-income countries, have high rates of VBAC [4]. It would be of interest to interview women from these countries about their experiences of VBAC.

Our metasynthesis shows that women's experiences were studied in relation to decision-making whether to give birth vaginally or with CS during the subsequent birth [2,13,20], experiences of the influence of health professionals on decision-making [30], and reason for trying a vaginal birth after a previous CS [18,20,31]. These aspects must be related to a maternity care where women have informed choice, and access to high quality care [32]. Further research is needed to see if informed choice is a problem for women when the information given is unclear. In this study, to be involved in decision-making about mode of delivery was found to be difficult but important. According to Cox, [14] Changing Childbirth in 1993 in UK gave women more choice over their maternity care, and it may be that this has led to many women making a ‘choice’ to have a repeat CS instead of VBAC [14], as has happened in other countries, with a resulting steep increase in CS-rate [32]. One could question why do women choose CS when VBAC is the best option, from an empirical evidence-base [10,11]. One answer according to our study is that women are *groping in the fog* in a context where vaginal birth is seen as a risky project and positive aspects of vaginal birth are mainly described by women and not the health care system.

The women had to confront serious risks mediated by health-professionals, and lack of information about the benefits of vaginal birth. These findings may be related to the provision of maternity care with a risk focus [33,34]. Our study shows that women experienced risk mediated by different percentages (20–70%) in relation to their individual risks [18,30], and as ‘odds’ being against them [18]. Information given to women should be derived from the most recent evidence. A woman with no other risk factors who was told that she might have a chance of *50/50* for a successful vaginal birth after CS had not been informed appropriately [30]. References from various studies conclude that women have a 74% chance for a successful VBAC if no further risk is obvious [7]. The risks that the women are informed about are uterine rupture [2,13], death of the child or mother or both [13,19], and the risk of ending up having another CS [2,13], and of being irresponsible and putting the baby at risk [19,30]. These results indicate that the women were well informed about potential risks, but perhaps not always accurately, and are not informed about the benefits of vaginal birth.

The results show that vaginal birth has several positive aspects, mainly described by the women. They felt they had their ‘own strong responsibility for giving birth vaginally’. Vaginal birth is experienced by women as good for the baby and the relationship and as a meaningful experience for them as women in line with birth as a life event described by Larkin et al. [35], and birth as an opportunity for women to gain an understanding of their strengths [36].

Limitations of the study are that three studies referred to the same group of data. All metasynthesis studies are in themselves three times removed from the participants’ lives [24]. We tried, however, to preserve the significance of the primary findings in the studies, and to remain close to them. This metasynthesis may complement the individual studies but they cannot replace them [23,26].

## Conclusion

Due to the rising CS-rate increasing numbers of women and health professionals have to decide mode of delivery in the subsequent birth. Vaginal birth is recommended as best practice for the majority of women, associated with lower maternal mortality than repeat CS, and less overall morbidity for mothers and babies. However, there are few studies about women's experiences of VBAC. This metasynthesis based on eight studies from an Anglo-American context, where informed choice is an option, raise the question of why women's experiences are not studied in other countries and maternity care settings. The study gives an understanding of how difficult VBAC is from women's perspectives. The women are *groping through the fog* and must have a strong sense of their own responsibility for giving birth vaginally since VBAC is mainly described by health professionals in relation to the risks involved. Women are well informed about these risks, but positive aspects of VBAC are mainly described by the women themselves. Giving birth vaginally is described as empowering, as best for the baby and as important in a life-perspective for them as women. In order to promote VBAC, more studies from different countries and maternity care settings are needed. Maternity care professionals must give women evidence-based information not only on risks but also on positive aspects of VBAC.

## Abbreviations

VBAC: Vaginal birth after Caesarean section ; CS: Caesarean section.

## Competing interests

The authors declare that they have no competing interest in the present research.

## Authors' contributions

IL, CB and MG participated in design of the study. All authors participated in data analysis, and drafted the manuscript. TB carried out a critical revision in relation to the method. All authors read and approved the final version.

## Pre-publication history

The pre-publication history for this paper can be accessed here:

http://www.biomedcentral.com/1471-2393/12/85/prepub
